# De novo development of JAK2V617F-positive polycythemia vera following allogeneic hematopoietic stem cell transplantation: a report of two rare cases

**DOI:** 10.48101/ujms.v131.14276

**Published:** 2026-10-01

**Authors:** Gulten Korkmaz

**Affiliations:** Department of Hematology, Ankara Bilkent City Hospital, Çankaya/Ankara, Turkey

**Keywords:** Polycythemia vera, allogeneic HSCT, JAK2, CML, MDS

## Abstract

**Introduction:**

Post-transplant erythrocytosis following allogeneic hematopoietic stem cell transplantation (allo-HSCT) is rare and has been reported mainly in patients with aplastic anemia and, less frequently, after transplantation for other hematologic conditions. Here, we present two cases of JAK2V617F-positive polycythemia vera (PV) developing after allo-HSCT performed for chronic myeloid leukemia (CML) and myelodysplastic syndrome (MDS).

**Case presentations:**

**Case 1:** The patient, who underwent allo-HSCT for the diagnosis of MDS. He was diagnosed with JAK2V617F-positive PV, 48 months after transplantation. Case 2: The patient with chronic-phase CML underwent allo-HSCT while in hematologic remission. Approximately 10 years later, the patient was diagnosed with JAK2V617F-positive PV.

**Discussion:**

Possible explanations for the development of a JAK2V617F mutation after allo-HSCT include the expansion of a pre-existing but clinically silent mutant clone in the donor, or the emergence of de novo mutations in donor or recipient cells as a result of proliferative stress, immunosuppression, or prior chemotherapy.

## New findings

We report two rare cases of JAK2V617F-positive polycythemia vera (PV) developing after allogeneic hematopoietic stem cell transplantation (allo-HSCT). Notably, this is the first reported case of PV occurring after allo-HSCT performed for chronic myeloid leukemia. In both patients, PV emerged under complete donor chimerism, highlighting the possibility of de novo clonal evolution following transplantation. The contrasting donor JAK2V617F statuses and the long latency periods observed in these cases provide novel insights into the mechanisms of post-transplant myeloproliferative neoplasms.

## Introduction

Allogeneic hematopoietic stem cell transplantation (allo-HSCT) is a potentially curative treatment modality for many malignant hematologic disorders. Erythrocytosis is defined by elevated hemoglobin (hgb) and hematocrit (HTC) levels due to increased red cell mass and can be either primary or secondary. Post-transplant erythrocytosis is a well-known complication observed in approximately 10–15% of kidney transplant recipients (1, 2). In contrast, erythrocytosis following allo-HSCT is rare and has been reported mainly in patients with aplastic anemia and, less frequently, after transplantation for other hematologic conditions (3–6). Cases in the literature include a patient with acute myeloid leukemia (AML) who developed polycythemia vera (PV) 5 years after chemotherapy without transplantation, a patient with myelodysplastic syndrome (MDS) who developed donor-derived JAK2V617F positivity following allo-HSCT, and a patient with AML who developed PV after autologous HSCT (7–9). However, JAK2V617F-positive PV developing after transplantation in a patient with chronic myeloid leukemia (CML) has not yet been reported. Myeloproliferative neoplasms (MPNs) are classified as Philadelphia (Ph) chromosome–positive CML and Ph-negative MPNs, including PV, essential thrombocythemia, and primary myelofibrosis. While CML is characterized by the BCR::ABL1 fusion gene (breakpoint cluster region::ABL proto-oncogene 1, non-receptor tyrosine kinase), Ph-negative MPNs typically harbor mutations in Janus kinase 2 (JAK2), calreticulin (CALR), or MPL proto-oncogene, thrombopoietin receptor (MPL), which are generally considered mutually exclusive (10). Nevertheless, concurrent expression of BCR::ABL1 translocation and JAK2 mutation has been reported in rare cases and was reviewed in 2024 (11). Here, we present two cases of JAK2V617F-positive PV developing after allo-HSCT performed for CML and MDS.

## Case presentations

### Case 1

A 46-year-old man, who had received multiple red blood cell transfusions for severe anemia at age 28, was referred to our center in November 2006 for further evaluation.

**Table 1:** Clinical characteristics of the two patients with post-transplant JAK2V617F-positive polycythemia vera.

Feature	Case 1	Case 2
Original diagnosis	MDS (RAEB-2)	CML (Chronic Phase)
Type of transplant	Allo-HSCT (MSD)	Allo-HSCT (MSD)
Time to PV diagnosis after Allo-HSCT	48 months (4 years) post-transplant	~120 months (10 years) post-transplant
New diagnosis	JAK2V617F-positive PV	JAK2V617F-positive PV
BCR::ABL1 at the time of PV diagnosis	Not evaluated	Negative
Hb / Hct at PV diagnosis	18.8 g/dL / 55.9%	18.7 g/dL / 56%
JAK2V617F status at PV diagnosis	Positive	Positive
Chimerism status	100% Donor	100% Donor
Donor sex	Male	Female
Donor JAK2V617F status	Negative	Unknown

MDS: Myelodysplastic Syndrome; RAEB-2: Refractory Anaemia With Excess Blasts-2; CML: Chronic Myeloid Leukemia; MSD: Matched Sibling Donor; Allo-HSCT: Allogeneic Hematopoietic Stem Cell Transplantation.

Complete blood count revealed: white blood cells (WBC): 2,100/mm^3^; absolute neutrophil count (ANC): 200/mm^3^; hgb level: 5.5 g/dL; mean corpuscular volume (MCV): 108 fL; and platelets (plt): 29,000/mm^3^. Peripheral smear showed macrocytosis and dysplastic neutrophils with hypogranulation and hyposegmentation, without an increase in blasts. Bone marrow aspirate and biopsy demonstrated 90% cellularity with marked trilineage dysplasia, 6% blasts, increased iron stores, and no ring sideroblasts. Cytogenetic analysis showed monosomy 7 and hypodiploidy (43, XY). A diagnosis of MDS- refractory anaemia with excess blasts-2 (RAEB-2) was made. On February 12, 2007, the patient underwent allo-HSCT from HLA-matched sibling donors (MRD), using a myeloablative conditioning (MAC) regimen. Neutrophil engraftment occurred on day +15 and platelet engraftment on day +20. No graft-versus-host disease (GVHD) developed during follow-up. Chimerism analysis at 1, 3, 6, and 12 months showed 100% donor chimerism. In March 2011, 48 months post-transplant, laboratory results revealed elevated Hgb (18.8 g/d) and HTC (55.9%) levels. Serum erythropoietin was suppressed, and the JAK2V617F mutation was detected. According to 2008 WHO criteria, the patient was diagnosed with PV. Repeat chimerism confirmed full donor origin, and the donor’s JAK2V617F test was negative. The patient was started on low-dose aspirin (100 mg/day) and periodic phlebotomy. At the time of writing, the patient remains stable without thrombotic events.

### Case 2

In April 2004, a 23-year-old male patient presented with abdominal pain. Due to splenomegaly and complete blood count results showing WBC: 400,000/mm^3^, Hgb: 14 g/dL, and Plt: 560,000/mm^3^, he was referred to the hematology department. Peripheral smear revealed granulocytosis with neutrophils and immature granulocytes, and bone marrow biopsy showed hypercellularity with myeloid and megakaryocytic proliferation. Cytogenetic analysis demonstrated a karyotype of 46, XY, t(9;22) [20]. JAK2V617F mutation was negative, and fluorescent in situ hybridization (FISH) analysis detected a BCR::ABL1 fusion in 99% of interphase nuclei, indicating a t(9;22)(q34;q11) translocation. A diagnosis of chronic-phase CML was made. Since imatinib was not yet available in our country at that time, hydroxyurea therapy was initiated. While in hematologic remission, the patient underwent allogeneic allo-HSCT from HLA-MRD on September 6, 2004, following a MAC regimen. Neutrophil engraftment occurred on day 14 and platelet engraftment on day 15. During follow-up, treatment was administered for grade 2 hepatic and grade 1 skin GVHD. At the 1st, 3rd, 6th, and 12th months post-transplant, full donor chimerism was confirmed. On March 23, 2013, approximately 10 years after allo-HSCT, laboratory tests revealed WBC: 11,610/mm^3^, Hgb: 18.7 g/dL, HTC: 56%, and Plt: 622,000/mm^3^. The spleen measured 15 cm. Real-time quantitative polymerase chain reaction (Q-PCR) was negative for BCR::ABL, while the JAK2V617F mutation test was positive at 6%. Erythropoietin was suppressed. Repeat chimerism testing confirmed full donor chimerism. The donor declined hospital evaluation; therefore, the donor’s JAK2V617F mutation status could not be assessed. Repeat bone marrow biopsy demonstrated hypercellularity for age with marked erythroid, granulocytic, and megakaryocytic proliferation and grade 1 reticulin fibrosis. Cytogenetic analysis revealed 46, XY [20], and FISH analysis was negative for BCR::ABL1 fusion. The patient was diagnosed with PV. Because of a history of coronary artery disease and coronary stent placement 1 year earlier, he was considered at high vascular risk. Hydroxyurea therapy was initiated, and he continued treatment with acetylsalicylic acid and ticagrelor prescribed by cardiology. Later, cardiology recommended discontinuation of ticagrelor. The patient continues treatment with hydroxyurea and aspirin. No further venous or arterial thrombosis has occurred.

## Discussion

After Allo-HCST, the recipient’s hematopoietic system is normally completely replaced by donor-derived cells. The detection of a JAK2V617F mutation after allo-HSCT may originate either from a newly acquired mutation in the recipient or from donor-derived cells. Donor cell–derived myeloid neoplasms (DDMN) have been previously reported. The proposed mechanisms include the expansion of a pre-existing subclinical mutant clone in the donor due to the new bone marrow microenvironment, or the induction of de novo mutations in donor cells triggered by factors such as proliferative stress, immunosuppression, or chemotherapy (12, 13). A case of donor-derived PV occurring 7 years after allo-HSCT performed for MDS was reported in 2008 (8). In our first case, the patient underwent allo-HSCT for MDS and was diagnosed with JAK2V617F-positive PV 4 years after transplantation. A case in which a patient diagnosed with AML underwent autologous HSCT and was later found to have JAK2V617F-positive PV 5 years afterward was first reported in 2007. In this case, retrospective Q-PCR analysis of archived samples showed that the JAK2V617F mutation, which had been negative at the time of AML diagnosis, became positive 4 years after AML treatment (9). In another patient who underwent autologous HSCT for AML, the development of JAK2V617F-positive ET 16.5 years later was reported (14). Furthermore, the development of JAK2V617F-positive PV following chemotherapy alone, without HSCT, has also been described in a patient with AML (7). These examples demonstrate that not only DDMN after allo-HSCT but also newly acquired mutations in the patients themselves can occur. Since the donor tested negative for the JAK2V617F mutation, we cannot definitively conclude that the mutation in our patient was donor-derived, as in the case reported by Van Pelt et al. (8) However, because the patient showed complete donor chimerism at the time of PV diagnosis, we believe that the mutation is more likely to represent a newly acquired donor-derived mutation rather than a new recipient-derived mutation.

In our second case, the patient underwent allo-HSCT for CML and was diagnosed with PV 10 years later. MPNs are classified as Ph chromosome–positive CML and Ph-negative MPNs. However, in the literature, cases have been reported in which patients with CML developed JAK2V617F positivity while in complete molecular remission following tyrosine kinase inhibitor therapy, as well as cases demonstrating concurrent or sequential positivity for JAK2V617F and BCR::ABL (15–17). However, to date, no case of PV developing after allo-HSCT performed for CML has been reported. In our patient, the JAK2V617F mutation was negative at the time of CML diagnosis but became positive at the time of PV diagnosis. Additionally, at the time PV was diagnosed, the patient was BCR::ABL-negative and exhibited complete donor chimerism. Because the donor declined to visit the hospital, the donor’s JAK2V617F mutation status remains unknown. Therefore, it cannot be determined whether the JAK2V617F mutation represents a newly acquired mutation in the recipient or a donor-derived mutation.

In conclusion, possible explanations for the development of a JAK2V617F mutation after allo-HSCT include the expansion of a pre-existing but clinically silent mutant clone in the donor, or the emergence of de novo mutations in donor or recipient cells as a result of proliferative stress, immunosuppression, or prior chemotherapy.
